# Potential hazard assessment of 6PPD-quinone in the context of ulcerative colitis: network toxicology, machine learning, transcriptomic analysis, and preliminary *In vivo* validation

**DOI:** 10.3389/fimmu.2026.1848350

**Published:** 2026-08-31

**Authors:** Jingyi Li, Xizhuang Gao, Yemin Xu, Lu Wang, Ying Zhu, Bin Deng

**Affiliations:** 1 Department of Gastroenterology, Northern Jiangsu People’s Hospital Affiliated to Yangzhou University, Yangzhou, Jiangsu, China; 2 Department of Functional Examination, Zaozhuang Municipal Hospital, Jining Medical University, Zaozhuang, Shandong, China

**Keywords:** 6PPD-quinone, adverse outcome pathway, machine learning, molecular dynamics, network toxicology, ulcerative colitis

## Abstract

**Background:**

The ubiquitous tire-derived pollutant 6PPD-quinone (6PPD-Q) poses potential systemic health risks, yet its toxicological impact on the intestinal tract, particularly in the context of ulcerative colitis (UC), remains largely unknown. This study aimed to investigate whether 6PPD-Q aggravates DSS-induced colitis and to identify candidate molecular events using integrative computational and experimental approaches.

**Methods:**

An integrative strategy combining network toxicology, machine learning, public bulk- and single-cell transcriptomic analyses, molecular simulations, and preliminary *in vivo* validation was applied. Molecular docking and molecular dynamics (MD) simulations were conducted to evaluate protein-ligand binding stability. For *in vivo* validation, a DSS-induced colitis mouse model was utilized. Furthermore, qRT-PCR and Western blot analyses were performed to evaluate colonic transcriptional alterations and tight-junction protein expression, respectively. Statistical analyses were conducted using Student’s t-test or one-way ANOVA, with P < 0.05 considered statistically significant.

**Results:**

Through multidimensional screening, we identified five core regulatory genes (*MAPKAPK2, ANXA5, CFB, NR1H4*, and *PLIN2*) potentially associated with 6PPD-Q and UC-related molecular alterations. Molecular docking and molecular dynamics simulations supported plausible predicted interactions between 6PPD-Q and the prioritized proteins, with MAPKAPK2 showing the most favorable docking score and a relatively stable simulated trajectory. *In vivo* experiments demonstrated that 6PPD-Q exposure significantly exacerbated DSS-induced colonic shortening, macroscopic lesions, and histopathological damage. Quantitative real-time polymerase chain reaction (qRT-PCR) analysis showed increased colonic mRNA expression of *Mapkapk2*, *Anxa5*, and *Cfb* and decreased expression of *Nr1h4* and *Plin2* in the DSS plus 6PPD-Q group, consistent with the directions predicted by the bioinformatics analyses. Based on these findings, a proposed Adverse Outcome Pathway (AOP) framework was constructed, linking 6PPD-Q exposure with candidate molecular targets, putative PI3K-Akt/MAPK signaling perturbations, intestinal immune dysregulation, and aggravated colonic injury.

**Conclusions:**

This study provides an integrative mechanistic framework for investigating the potential intestinal effects of 6PPD-Q under inflammatory conditions. By integrating computational target prioritization with *in vivo* phenotypic and transcriptional evidence, the study identifies candidate molecular events that may contribute to 6PPD-Q-exacerbated intestinal inflammation and provides testable hypotheses for subsequent toxicological investigation.

## Graphical Abstract

**Figure:**
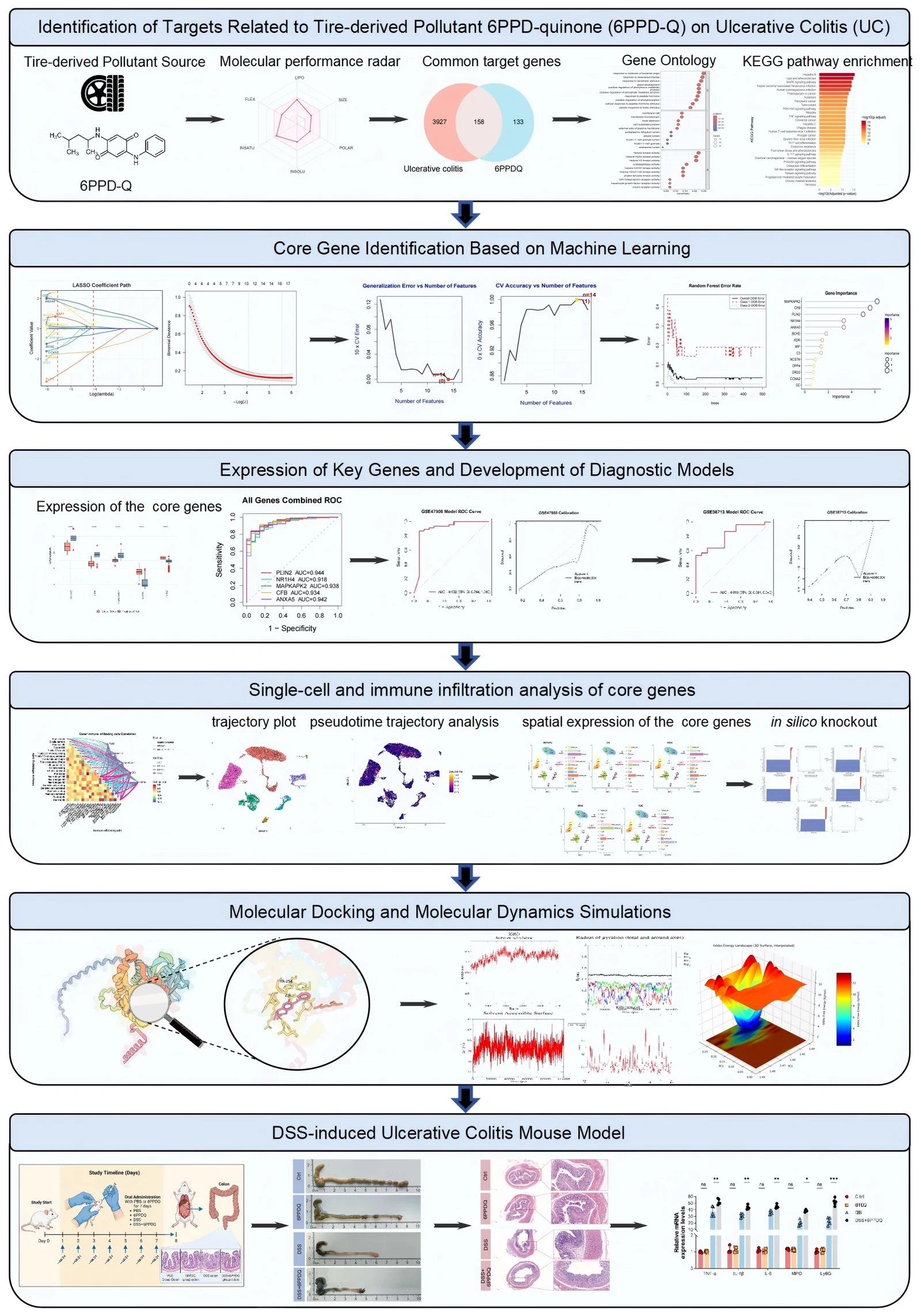

## Highlight

- 6PPD-Q exacerbated DSS-induced colitis and enhanced local inflammatory responses.
- Machine learning prioritized five candidate genes associated with 6PPD-Q-exacerbated colonic injury.
- Molecular simulations supported a plausible and relatively stable predicted interaction between 6PPD-Q and MAPKAPK2.
- A four-group mouse experiment demonstrated that 6PPD-Q aggravated DSS-induced colitis and produced transcriptional changes consistent with the computational predictions.
- A proposed adverse outcome pathway provides a testable framework for 6PPD-Q-associated intestinal injury.

## 1 Introduction

In recent years, the adverse effects of environmental toxins on human health have garnered increasing attention. With the rapid development of the automotive industry, global demand for rubber tires has surged significantly. The large-scale production and use of tires have resulted in substantial releases of tire-related chemical pollutants into the environment (1). Among these, N-(1,3-dimethylbutyl)-N’-phenyl-p-phenylenediamine (6PPD) and its oxidation conversion product N-(1,3-dimethylbutyl)-N’-phenyl-p-phenylenediamine quinone (6PPD-Q)—widely used as antioxidants in rubber tires—have emerged as critical contaminants of concern (2–4). Monitoring studies indicate that 6PPD-Q can be detected in various environmental media, including atmospheric particulate matter, road dust, soil, surface runoff, and surface water. It accumulates in food sources such as honey, fish, and vegetables, as well as in human urine samples. This suggests that human exposure, particularly through ingestion, is widespread and unavoidable (5, 6). Toxicological evidence indicates that 6PPD-Q can induce adverse effects including hepatotoxicity, immune-mediated liver injury, and neuro- and reproductive toxicity, and is considered to potentially pose systemic risks to human health (7–11). However, despite the gastrointestinal tract being a primary contact site for ingested contaminants, the pharmacokinetic characteristics and toxicological mechanisms of 6PPD-Q in the digestive system—particularly within the colonic mucosa—remain largely unexplored.

Ulcerative colitis (UC) is a chronic, recurrent, non-specific inflammatory bowel disease primarily affecting the mucosa of the colon and rectum. Its development is believed to result from the combined effects of genetic susceptibility, mucosal immune dysfunction, and intestinal environmental factors (12). In recent years, numerous epidemiological and experimental studies have suggested that environmental pollutants—such as fine particulate matter PM2.5, polycyclic aromatic hydrocarbons, and persistent organic pollutants—can exacerbate intestinal inflammation by disrupting the intestinal epithelial barrier, inducing oxidative stress, and activating mucosal immunity. This indicates the significant role of exogenous chemicals in the onset and recurrence of ulcerative colitis (13–15).Unlike PM2.5, which represents a heterogeneous mixture of particles and particle-associated chemicals, 6PPD-Q is a structurally defined quinone transformation product of the tire antioxidant 6PPD. This distinction enables the investigation of chemical-specific molecular interactions and signaling perturbations. Previous studies in otherwise healthy mice demonstrated that oral 6PPD-Q exposure predominantly impaired jejunal and ileal barrier integrity and induced cannabinoid receptor-associated inflammatory responses, whereas marked colonic injury was not observed. These observations raise an important disease-susceptibility question: whether 6PPD-Q can aggravate tissue injury when the colonic epithelial barrier and mucosal immune homeostasis are already compromised, as occurs in UC. Accordingly, the present study focuses specifically on the interaction between 6PPD-Q exposure and pre-existing experimental colitis. However, compared to research exploring the toxic mechanisms affecting the liver, cardiovascular system, and reproductive system, studies on whether and how 6PPD-Q contributes to the onset and progression of ulcerative colitis remain scarce. This limitation has hindered a systematic assessment of the intestinal health hazards posed by this emerging contaminant.

From an inflammatory immunological perspective, one of the core pathological features of UC is an imbalance in the intestinal mucosal immune response. This imbalance encompasses disruption of the epithelial barrier, abnormal activation of dendritic cells and macrophages, and a Th1/Th17 cell-mediated cascade amplification of inflammatory cytokines (16–19). Previous studies have demonstrated that multiple environmental toxins can activate signaling pathways such as NF-κB, MAPK, and JAK/STAT, thereby upregulating inflammatory mediators including TNF-α, IL-6, IL-1β, and IL-17. This amplifies mucosal inflammatory responses and promotes the sustained activity or recurrence of ulcerative colitis (17, 20). Given the immunotoxicity and proinflammatory effects exhibited by 6PPD-Q in other target organs, it is reasonable to speculate that this compound may contribute to the immune-inflammatory imbalance in ulcerative colitis by interfering with mucosal-associated signaling pathways and promoting immune cell infiltration. Nevertheless, several important gaps remain in the current understanding of 6PPD-Q intestinal toxicity. First, previous studies have predominantly examined otherwise healthy organisms and have therefore not determined whether pre-existing intestinal inflammation increases susceptibility to 6PPD-Q. Second, most studies have focused on general intestinal phenotypes, such as barrier permeability, oxidative stress, inflammatory cytokine production, or metabolic disruption, without systematically resolving UC-associated molecular networks and cell-type-specific responses. Third, the relationship between computationally predicted 6PPD-Q targets, human UC transcriptomic alterations, and experimentally aggravated colitis has not been comprehensively evaluated. These limitations motivated the present integrative investigation combining network toxicology, machine learning, bulk and single-cell transcriptomic analyses, structural simulations, and *in vivo* phenotypic validation.

Network toxicology, combined with machine learning and multidimensional bioinformatics analysis, provides new tools for deciphering the potential links between complex environmental pollutants and chronic inflammatory diseases. On one hand, by leveraging *in vitro* target prediction and disease database integration, a candidate gene set co-associated with 6PPD-Q and ulcerative colitis can be systematically identified. Gene Ontology (GO) and Kyoto Encyclopedia of Genes and Genomes (KEGG) enrichment analysis can then be performed to elucidate the biological processes and key signaling pathways involved. On the other hand, machine learning algorithms such as LASSO regression and SVM-RFE can be employed to identify core genes most closely associated with UC from a large pool of candidate genes. These targets can then be validated at the cellular and immune microenvironment levels by integrating transcriptomic, immune infiltration analysis, and single-cell RNA sequencing data. Furthermore, molecular docking and molecular dynamics simulations can characterize the binding patterns and interaction stability between 6PPD-Q and key proteins at the molecular level, providing structural and energetics-based evidence for constructing an Adverse Outcome Pathway (AOP) for ulcerative colitis that links chemical exposure to molecular initiation events, key events, and adverse outcomes.

Against this backdrop, this study aims to investigate the potential toxicological mechanisms of 6PPD-Q on ulcerative colitis through an integrative strategy of computational network toxicology and *in vivo* experimental validation. First, machine learning and multidimensional bioinformatics (including single-cell RNA sequencing) were utilized to prioritize candidate targets and immune-inflammatory processes potentially associated with predicted 6PPD-Q-related targets and UC-related molecular alterations. Second, molecular docking and molecular dynamics simulations were performed to evaluate predicted interaction patterns between 6PPD-Q and the prioritized proteins. Crucially, an *in vivo* DSS-induced UC mouse model was established to validate the exacerbating effects of 6PPD-Q on colonic mucosal injury, and the transcriptional changes of the prioritized candidate genes were further evaluated using qRT-PCR. Ultimately, this multi-tiered approach allows us to propose a hypothesis-generating Adverse Outcome Pathway (AOP) framework. This study aims to elucidate the potential role of emerging tire wear pollutants in promoting inflammatory bowel diseases at the mechanistic level, providing scientific evidence for environmental health risk assessments of ulcerative colitis and the development of relevant prevention and control strategies.

## 2 Methods

### 2.1 Chemical structure and ADME analysis of 6PPD-Q

The structural information and SMILES data for 6PPD-Q were obtained from the PubChem database (https://pubchem.ncbi.nlm.nih.gov/). The SwissADME web tool was used to predict the ADME properties of 6PPD-Q, including key parameters such as gastrointestinal absorption (GI absorption), blood-brain barrier permeability (BBB permeant), cytochrome P450 inhibition (CYP), and skin permeability (Log Kp).

### 2.2 Identification of potential targets for 6PPD-Q

The SwissTargetPrediction platform (http://swisstargetprediction.ch/) was employed to predict its potential biological targets, with species restricted to Homo sapiens. This analysis was integrated with the Comparative Toxicogenomics Database (CTD, http://ctdbase.org), SEA (http://sea.bkslab.org/), TargetNet (http://targetnet.scbdd.com/), and PharmMapper (https://www.lilab-ecust.cn/pharmmapper/submitfile.html) to obtain supplementary target information. After removing duplicate entries from the obtained results, the merged dataset was organized into a comprehensive list of candidate targets associated with 6PPD-Q.

### 2.3 Identification of ulcerative colitis-related targets

Genes associated with ulcerative colitis were identified through keyword searches in the GeneCards database (https://www.genecards.org/) and the Online Mendelian Inheritance in Man database (OMIM) (https://omim.org/). The gene sets obtained from both databases were merged, screened, and deduplicated to generate a final list of candidate targets potentially involved in the pathogenesis of ulcerative colitis.

### 2.4 Functional pathway analysis of target genes

We performed enrichment analysis of Gene Ontology (GO) and Kyoto Encyclopedia of Genes and Genomes (KEGG) to explore molecular pathways associated with potential target genes of 6PPD-Q-induced ulcerative colitis. GO terms were categorized into biological processes (BP), cellular components (CC), and molecular functions (MF). Only items with adjusted p-values < 0.05 were considered statistically significant.

### 2.5 Core target prioritization by machine-learning algorithms

We employed three machine-learning methods—least absolute shrinkage and selection operator (LASSO) logistic regression, support vector machine–recursive feature elimination (SVM-RFE), and random forest (RF)—to prioritize candidate genes potentially linking predicted 6PPD-Q-associated targets with UC-related transcriptional alterations. LASSO applies L1 regularization to perform coefficient shrinkage and variable selection, SVM-RFE sequentially eliminates relatively uninformative features according to their contribution to classification performance, and RF provides an ensemble tree-based approach for evaluating nonlinear feature contributions and ranking variable importance.

A sequential feature-prioritization strategy was implemented using the expression profiles of the 158 candidate genes in the GSE87473 cohort. UC and normal mucosal samples were encoded as a binary outcome. Before model construction, gene-expression values were standardized by centering each feature to a mean of zero and scaling it to unit variance. A fixed random seed was used to ensure reproducibility.

LASSO logistic regression was first performed using the glmnet package in R with family = “binomial”, alpha = 1, standardize = TRUE, nfolds = 3, and type.measure = “deviance”. The regularization parameter was selected using lambda.min, corresponding to the value associated with the minimum mean cross-validated binomial deviance. Sixteen genes with nonzero coefficients at the selected λ value were retained for subsequent analysis.

SVM-RFE was then applied to the 16 LASSO-retained genes using the caret package. A linear support vector machine classifier was constructed with a cost parameter of C = 1. Recursive feature elimination was evaluated using 10-fold cross-validation, with classification accuracy used as the model-selection metric. Candidate subset sizes ranging from 1 to 16 genes were assessed, and the subset containing 14 genes produced the highest cross-validated accuracy and the lowest corresponding generalization error.

Finally, an RF classification model was constructed from the 14 SVM-RFE-retained genes using the randomForest package. The model included 500 trees (ntree = 500), and the number of variables randomly sampled at each split was set to mtry = 3, corresponding to the square root of the number of input features rounded down. Variable importance was assessed using the mean decrease in Gini impurity (MeanDecreaseGini), while the out-of-bag error rate was monitored across the number of trees to assess model stabilization. The ten highest-ranked genes were visualized, and the five genes with the highest importance scores—MAPKAPK2, CFB, PLIN2, NR1H4, and ANXA5—were carried forward as the prioritized candidate targets.

This sequential machine-learning workflow was used for exploratory candidate-gene prioritization and provided the basis for subsequent transcriptomic, single-cell, structural, and *in vivo* analyses. The internally derived feature rankings and discriminatory performance should not be interpreted as evidence of an independently validated clinical biomarker model.

### 2.6 Expression analysis and external validation of the five-gene model

The expression levels of the five selected genes were evaluated in the GSE87473 discovery cohort, which included transcriptomic profiles of UC and normal intestinal mucosal samples. Differences in gene expression between the two groups were assessed, and receiver operating characteristic (ROC) curves were generated to evaluate the individual discriminatory performance of each gene. A five-gene logistic regression model incorporating MAPKAPK2, CFB, PLIN2, NR1H4, and ANXA5 was developed using the GSE87473 cohort. The model intercept and regression coefficients derived from GSE87473 were fixed and subsequently applied to two independent external cohorts, GSE47908 and GSE38713. Detailed information on the platforms, tissue types, sample composition, group definitions, and references for all datasets is provided in **Table 1**.

**Table 1:** Details of the UC datasets used in this study.

GEO accession	Platform	Tissue	UC	Control	Reference	Group
GSE87473	GPL13158	Colonic mucosa	106	21	PMID: 29401083	Discovery cohort
GSE47908	GPL570	Colonic mucosa	45	15	PMID: 25358065	External validation cohort 1
GSE38713	GPL570	Colonic mucosa	30	13	PMID: 23135761	External validation cohort 2
GSE214695	GPL18573	Colonic mucosa	6	6	PMID: 37495570	scRNA-seq cohort

Abbreviations: UC, ulcerative colitis; GEO, Gene Expression Omnibus; scRNA-seq, single-cell RNA sequencing.

### 2.7 Assessment of immune cell infiltration

Immune-cell infiltration in UC and normal mucosal tissues was estimated using the CIBERSORT algorithm based on the normalized expression matrix of the GSE87473 cohort. The LM22 leukocyte signature matrix was used as the reference expression profile to estimate the relative proportions of 22 human immune-cell subsets. Differences in the estimated immune-cell fractions between UC and normal mucosal samples were evaluated using the Wilcoxon rank-sum test. Spearman’s rank correlation analysis was used to assess correlations among immune-cell subsets and associations between the expression levels of the five prioritized genes and the estimated immune-cell fractions.

### 2.8 Single-cell transcriptomic analysis of ulcerative colitis

Single-cell RNA-sequencing data were obtained from the Gene Expression Omnibus series GSE214695. The complete series contains colonic mucosal samples from six healthy controls, six patients with active ulcerative colitis, and six patients with active Crohn’s disease. The sample was generated using the 10x Genomics single-cell 3′ platform and sequenced using the Illumina NextSeq 500 platform. Initial demultiplexing, barcode processing, and gene counting were performed using Cell Ranger v3.1 with the GRCh38.p10 reference genome. The processed gene-expression matrix was analyzed using the Seurat R package. After cell-level quality control, normalization, and scaling, the top 2,500 highly variable genes were selected for principal component analysis. UMAP was used for nonlinear dimensionality reduction and visualization, and broad cell identities were assigned according to canonical marker-gene expression profiles. The single-cell analysis was used to characterize cellular heterogeneity and the cell-type-associated expression of the prioritized genes within an active-UC tissue context rather than to identify 6PPD-Q-induced changes. To exploratively characterize the relative transcriptional-state organization of the annotated cell populations, principal-graph and pseudotime analyses were conducted using the Monocle algorithm. Cells were computationally ordered along an inferred principal graph in reduced-dimensional transcriptomic space. Because the analysis included populations from distinct developmental lineages, the resulting ordering was interpreted descriptively and was not considered evidence of lineage differentiation, transdifferentiation, chronological progression, or direct cell-type conversion. In silico perturbation analyses were performed using the scTenifoldKnock R package within the cell populations showing relatively high expression of the corresponding prioritized genes. The analysis was used to predict potential downstream gene-regulatory-network alterations following computational suppression of each target. These results were considered hypothesis-generating and were not interpreted as experimental perturbation validation or evidence of causal regulation.

### 2.9 Molecular docking

To further investigate the interaction between 6PPD-Q and its core target protein, molecular docking software was employed to predict the binding mode and binding energy between 6PPD-Q and its target protein. The three-dimensional structures of 6PPD-Q and the target protein were obtained from PubChem and UniProt, respectively. Molecules were preprocessed using PYMOL software (version 3.10) by removing water molecules, adding hydrogen atoms, and calculating charges. AutoDock Tools software prepared the receptor and ligand files and set the docking box to define the binding site. Docking was performed using AutoDock Vina, visualized with PYMOL, and analyzed to determine the binding mode and affinity of 6PPD-Q with its core target.

### 2.10 Molecular dynamics simulation

To evaluate the dynamic stability of the complexes, 100 ns molecular dynamics (MD) simulation was performed using GROMACS 2022 software. The AMBER ff14SB force field was chosen for the protein, and the GAFF2 force field was used for the ligand. The TIP3P water model was selected to solvate the protein-ligand system in a cubic box with a periodic boundary of 1.2 nm. The Particle Mesh Ewald (PME) method was used to deal with long-range electrostatic interactions. Following energy minimization, 100 ps of NVT (isothermal-isochoric) ensemble equilibration and 100 ps of NPT (isothermal-isobaric) ensemble equilibration were performed. Both van der Waals and Coulomb interactions were calculated using 1.0 nm cutoff values. Finally, the system was simulated at a constant temperature (310 K) and constant pressure (1 bar) for a total of 100 ns.RMSD convergence was operationally assessed as the establishment of a sustained plateau without progressive directional drift during the later phase of the trajectory and was interpreted together with the radius of gyration, solvent-accessible surface area, root-mean-square fluctuation, protein–ligand hydrogen-bond dynamics, and the free-energy landscape.

### 2.11 Animal studies

#### 2.11.1 Mice

Wild-type (WT) mice (C57BL/6) were maintained in a specific pathogen-free environment with filtered air, sterile water, and autoclaved feed. Male mice aged 8 to 10 weeks and weighing 20 to 25 grams were used in the experiments. All animal experiments were reviewed and approved by the Institutional Animal Care and Use Committee of Yangzhou University.

#### 2.11.2 DSS-induced mouse colitis model

WT mice were administered 2.5% DSS (molecular weight 36,000–50,000; MP Biomedicals, Solon, OH, USA) in drinking water for 7 days. Mice received daily oral administration of 6PPD-Q at 0.1 mg/kg/day (CAS No. 2754428-18-5; purity ≥99%; MedChemExpress, Shanghai, China) or an equivalent volume of vehicle.

The oral route was selected to model gastrointestinal exposure, an important pathway through which humans may encounter environmental contaminants in food and water. The administered dose of 0.1 mg/kg/day, equivalent to 100 μg/kg/day, was chosen with reference to a previous murine intestinal toxicity study in which adult mice were orally exposed to 6PPD-Q at 0.1, 1, 10, and 100 μg/kg/day for 21 days. That study demonstrated dose-dependent intestinal barrier injury, with inflammatory responses particularly evident at 10 and 100 μg/kg/day. Accordingly, the present dose represents the upper end of a previously reported low-dose oral exposure range and was used as a literature-supported proof-of-concept dose to determine whether 6PPD-Q aggravates colonic injury under DSS-induced inflammatory conditions.

The mice were randomly divided into four groups: the vehicle-treated control group, the 6PPD-Q group, the DSS group, and the DSS plus 6PPD-Q group, with five mice in each group. Clinical indicators of acute colitis, including body weight, stool consistency, fecal bleeding, and survival, were assessed daily. On day 7, mice were anesthetized with sodium pentobarbital (50 mg/kg, intraperitoneally) and euthanized by cervical dislocation. Colon tissues were collected for measurement of colon length, histopathological examination, and molecular analysis.

#### 2.11.3 Histopathological examination (H&E staining)

For histological assessment, intestinal tissue was fixed overnight at 4 °C in 4% paraformaldehyde solution. Following dehydration, the tissue was embedded in paraffin and sectioned at 5-micron thickness. Sections underwent hematoxylin-eosin (H&E) staining according to standard operating procedures, and histopathological alterations were observed and documented under a light microscope.

#### 2.11.4 Quantitative real-time polymerase chain reaction

Total RNA was extracted from cultured cells or tissues using TRIzol reagent (Invitrogen, California, USA). Reverse transcription was performed using the PrimeScript™ II One-Step cDNA Synthesis Kit (Takara, Beijing, China). Quantitative reverse transcription PCR was conducted using the CFX96 Real-Time PCR Detection System (Bio-Rad, California, USA). To assess relative gene expression levels, glyceraldehyde-3-phosphate dehydrogenase (GAPDH) served as the normalization control. Relative colonic mRNA expression levels of *Anxa5, Cfb, Mapkapk2, Plin2, Nr1h4*, *Tnf-α, IL1-β, IL-6, Mpo*, and *Ly6g* were calculated using the 2^−ΔΔCt method. The forward and reverse primer sequences for all ten target genes and the reference gene are provided in **Supplementary Table 1**.

#### 2.11.5 Western blot analysis

Colonic tissues were lysed in radioimmunoprecipitation assay buffer supplemented with protease and phosphatase inhibitors. Total protein concentrations were determined using a bicinchoninic acid protein assay. Equal amounts of protein were separated by sodium dodecyl sulfate–polyacrylamide gel electrophoresis and transferred onto polyvinylidene fluoride membranes. After blocking, the membranes were incubated overnight at 4 °C with primary antibodies against ZO-1, claudin-1, occludin, and β-actin. The membranes were subsequently incubated with the corresponding horseradish peroxidase-conjugated secondary antibodies, and protein bands were visualized using an enhanced chemiluminescence detection system. Band intensities were quantified using ImageJ software. Western blot analysis was performed using 5 independent biological samples per group.

### 2.12 Construction of a proposed hypothesis-generating AOP framework

A proposed hypothesis-generating adverse outcome pathway framework was developed to organize the different levels of evidence generated in this study. Predicted interactions or perturbations of 6PPD-Q with the prioritized candidate targets were considered putative initiating interactions involving the five candidate targets. Enrichment of PI3K-Akt/MAPK-related signaling, inflammatory responses, immune dysregulation, and epithelial-barrier-associated injury were organized as candidate key events, while aggravated DSS-induced colitis was considered the adverse outcome under the tested experimental conditions. Computational predictions, disease-contextual evidence from public UC transcriptomic datasets, and direct experimental findings from the animal study were distinguished according to their respective evidence levels. This framework was intended to generate testable mechanistic hypotheses rather than to establish a validated causal AOP.

## 3 Results

### 3.1 Network toxicology analysis of 6PPD-Q-related targets in ulcerative colitis

**Figure 1:**
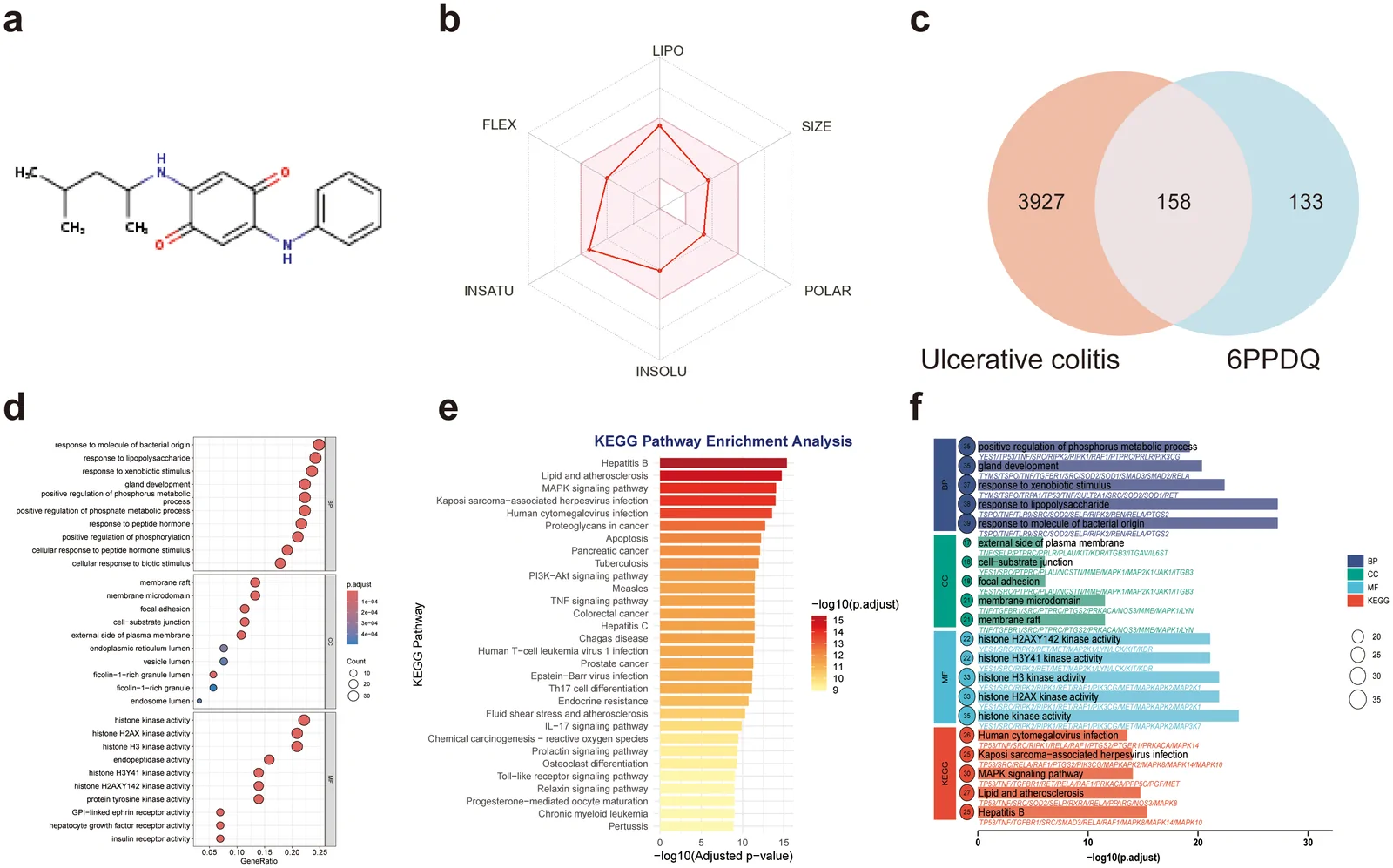
Identification of key candidate targets and biological functions of 6PPD-Q in ulcerative colitis (UC). **(a)** The two-dimensional (2D) chemical structure of 6PPD-Q. **(b)** Summary of the physicochemical and ADME properties of 6PPD-Q predicted by SwissADME. **(c)** Venn diagram showing the overlap between 6PPD-Q-associated and UC-associated targets; the shared genes represent the primary candidate targets of 6PPD-Q in the UC context. **(d)** Bubble chart displaying the Gene Ontology (GO) enrichment analysis of the identified candidate target genes. **(e)** Bar chart depicting the Kyoto Encyclopedia of Genes and Genomes (KEGG) pathway enrichment analysis. **(f)** Summary bar chart integrating the key GO and KEGG pathway enrichment results for the candidate targets.

Network toxicology provides an effective framework for elucidating the molecular mechanisms and potential targets of environmental toxins. Using this approach, we investigated the pathogenic role of 6PPD-Q in UC. The two-dimensional structure of 6PPD-Q was downloaded from the PubChem database and visualized (**Figure 1a**). We conducted a comprehensive ADME/toxicology analysis of the physicochemical and pharmacokinetic properties of 6PPD-Q using the specialized database SwissADME. The bioavailability radar indicated favorable lipophilicity and high gastrointestinal absorption, suggesting that ingested 6PPD-Q is highly bioavailable and capable of penetrating the intestinal epithelial barrier to exert localized and systemic toxicological effects (**Figure 1b** and **Supplementary Table 2**). To systematically identify potential pharmacological and toxicological targets of 6PPD-Q, data were integrated from five major databases: SwissTargetPrediction, CTD, SEA, TargetNet, and PharmMapper. After merging and deduplicating across the five toxicology databases, a total of 291 predicted targets for 6PPD-Q were identified. Simultaneously, after removing duplicate entries, 4,085 non-redundant UC-related genes were retrieved from GeneCards and OMIM. To visualize the intersection of these two gene sets, a Venn diagram was constructed, identifying 158 overlapping genes. These genes are considered potential targets for 6PPD-Q in promoting the pathogenesis of UC (**Figure 1c**).

### 3.2 Functional enrichment analysis of 6PPD-Q-related ulcerative colitis targets

To further investigate the potential mechanisms of 6PPD-Q in UC, we performed functional annotation of 158 overlapping target genes through GO and KEGG pathway analysis, providing deeper insights into the biological processes and signaling pathways associated with these targets. Enrichment analysis revealed that genes jointly targeted by 6PPD-Q and UC primarily participate in cellular responses to biological/bacterial components and peptide hormones, immune-inflammatory responses, and phosphorylation-related signaling regulation. These genes are also closely associated with membrane raft organization, cell adhesion structures, and receptor tyrosine kinase activity (**Figure 1d**). Furthermore, KEGG pathway analysis demonstrated that these intersecting genes were significantly enriched in key inflammatory and immune signaling networks, particularly the MAPK, PI3K-Akt, TNF, IL-17, and Toll-like receptor signaling pathways. They were also implicated in several viral infection and lipid metabolism-related pathways (e.g., Hepatitis B and atherosclerosis). Notably, mucosal inflammation-associated pathways, such as the MAPK cascade, exhibited the most pronounced enrichment, highlighting their central regulatory role in 6PPD-Q-induced UC exacerbation (**Figure 1e**). Taken together, the combined GO and KEGG analyses strongly suggest that 6PPD-Q exposure contributes to UC pathogenesis by jointly regulating these critical signaling networks, thereby driving the cascade amplification of inflammatory responses, remodeling the intestinal immune microenvironment, and potentially initiating inflammation-driven pathological processes (**Figure 1f**).

### 3.3 Core gene identification based on machine learning

**Figure 2:**
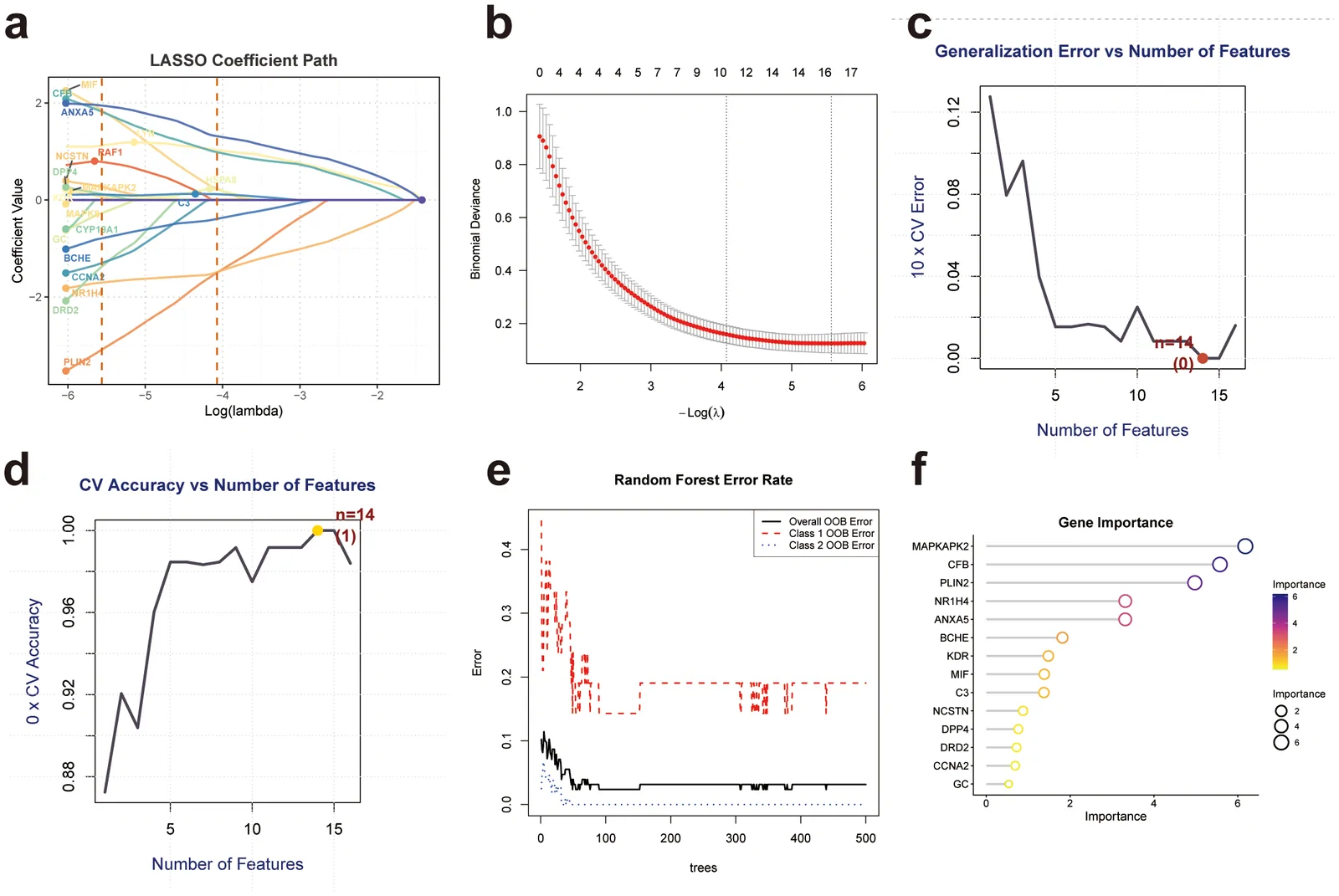
Machine-learning-based prioritization of candidate genes associated with 6PPD-Q and ulcerative colitis. **(a)** LASSO coefficient profiles of the candidate genes. **(b)** Three-fold cross-validation curve for selecting the regularization parameter; lambda.min was used, resulting in 16 genes with nonzero coefficients. **(c)** Generalization error across different SVM-RFE feature-subset sizes. **(d)** Ten-fold cross-validated classification accuracy across feature-subset sizes, with the optimal subset containing 14 genes. **(e)** Out-of-bag error rate of the random forest model across 500 trees. **(f)** Random-forest feature-importance ranking based on mean decrease in Gini impurity, from which the five highest-ranked genes were selected for subsequent analyses.

To identify key genes associated with 6PPD-Q-induced ulcerative colitis (UC), three machine learning algorithms—LASSO, SVM, and Random Forest (RF)—were applied to 158 candidate genes related to 6PPD-Q exposure and UC. Among these genes, LASSO logistic regression with 3-fold cross-validation identified 16 potential core regulatory factors (**Figures 2a, b**). SVM-RFE classification further refined the LASSO-retained feature set and identified an optimal subset of 14 genes based on cross-validated classification accuracy (**Figures 2c, d**). Based on this, RF identified ten top-ranked genes, with the top five genes designated as core targets (*MAPKAPK2, CFB, PLIN2, NR1H4, ANXA5*) (**Figures 2e, f**).

### 3.4 Expression patterns and individual discriminatory performance of the five selected genes

**Figure 3:**
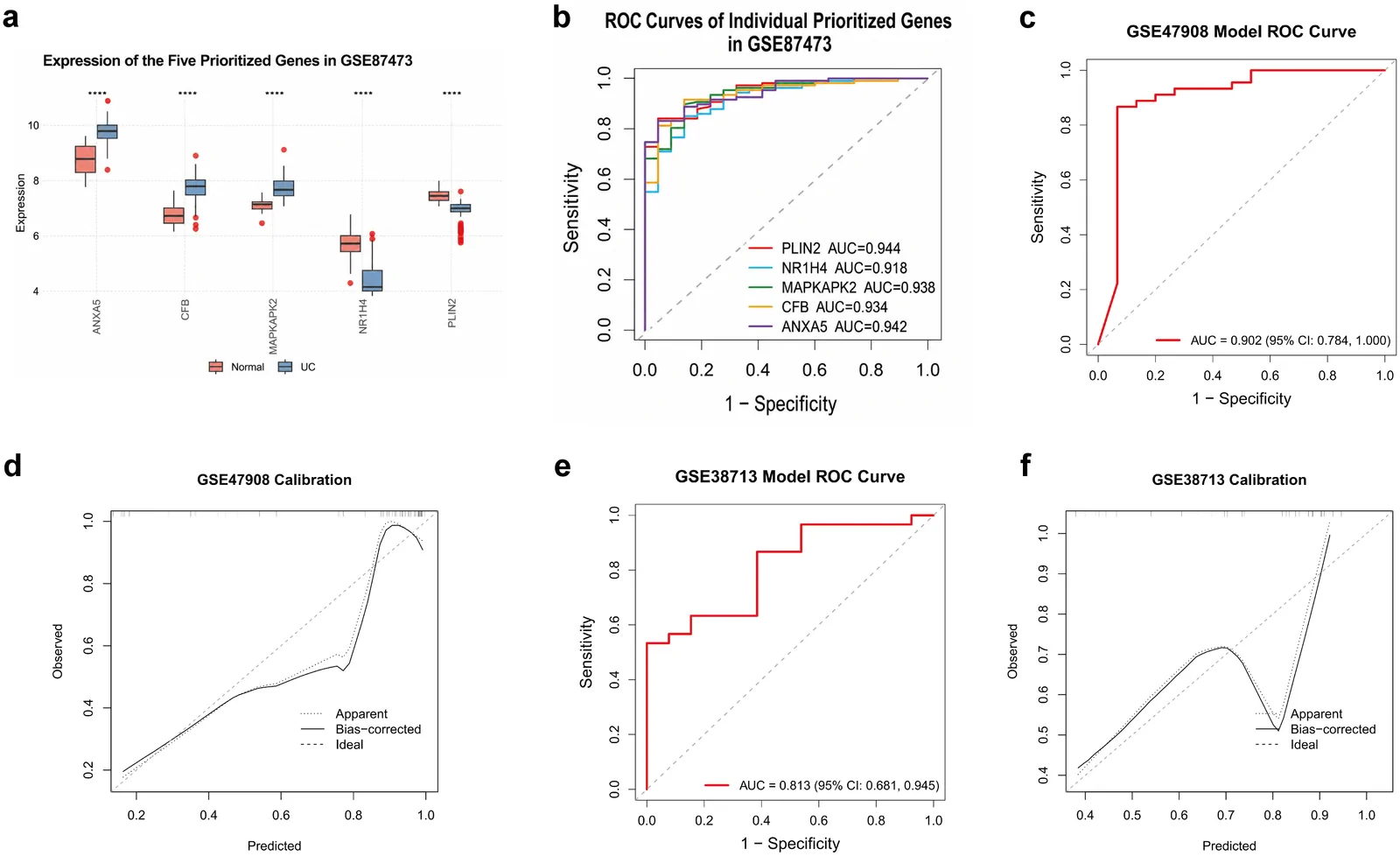
Expression patterns and diagnostic performance of the five prioritized genes in the discovery and external-validation cohorts. **(a)** Expression levels of ANXA5, CFB, MAPKAPK2, NR1H4, and PLIN2 in normal and ulcerative colitis mucosal samples from the discovery cohort GSE87473. **(b)** Receiver operating characteristic curves evaluating the individual discriminatory performance of the five prioritized genes in GSE87473. **(c)** Receiver operating characteristic curve of the five-gene model in the external-validation cohort GSE47908. **(d)** Calibration curve of the five-gene model in GSE47908. **(e)** Receiver operating characteristic curve of the five-gene model in the external-validation cohort GSE38713. **(f)** Calibration curve of the five-gene model in GSE38713.

The expression patterns of the five selected genes were evaluated in the GSE87473 discovery cohort. ANXA5, CFB, and MAPKAPK2 were significantly upregulated in UC mucosal samples compared with normal mucosal samples, whereas PLIN2 and NR1H4 were significantly downregulated (**Figure 3a**). Individual ROC analysis yielded AUC values of 0.942 for ANXA5, 0.934 for CFB, 0.938 for MAPKAPK2, 0.944 for PLIN2, and 0.918 for NR1H4 (**Figure 3b**). These findings demonstrate disease-associated expression patterns and individual discriminatory potential of the five genes in the GSE87473 cohort. Because the public transcriptomic dataset did not contain individual 6PPD-Q exposure information, these results provide UC-related disease-contextual evidence but do not independently establish exposure-induced dysregulation or direct toxicological mediation.

### 3.5 External validation of the five-gene model

The fixed five-gene model derived from the GSE87473 discovery cohort was evaluated in two independent external cohorts without additional feature selection or re-estimation of the original gene coefficients. In GSE47908, the model achieved an AUC of 0.902 (95% CI: 0.784–1.000), indicating good discriminatory performance (**Figure 3c**). In GSE38713, the model yielded an AUC of 0.813 (95% CI: 0.681–0.945), demonstrating retained but comparatively lower discrimination in a second independent cohort (**Figure 3e**).

Calibration analysis showed dataset-dependent agreement between predicted probabilities and observed outcomes, with deviations in some predicted-probability ranges, particularly in GSE38713 (**Figures 3d, f**). Collectively, these findings provide preliminary evidence that the five-gene model retains discriminatory performance across independent public datasets.

### 3.6 Correlation between core genes and immune cell infiltration

**Figure 4:**
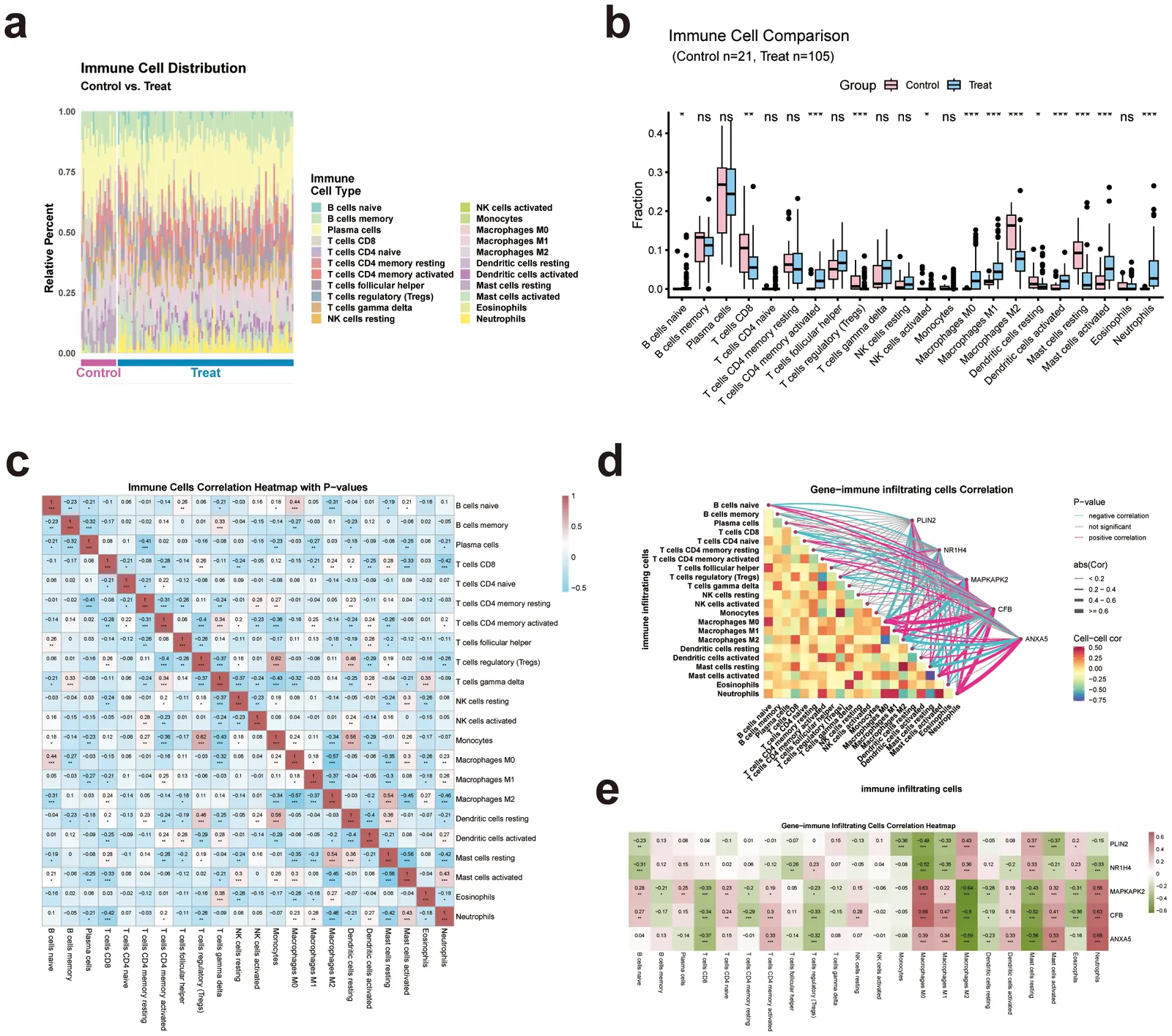
Analysis of immune cell infiltration in UC and normal intestinal mucosa based on the GSE87473 dataset. **(a)** Relative distribution of the 22 distinct immune cell subpopulations in UC patients and healthy controls, evaluated using the CIBERSORT algorithm. **(b)** Quantitative comparison of the infiltration levels of the 22 immune cell types between the UC and normal intestinal mucosa groups. Statistical significance is indicated as follows: ns, not significant; *P < 0.05, **P < 0.01, ***P < 0.001. **(c)** Correlation heatmap illustrating the interaction patterns among different immune cell subsets in the UC microenvironment. **(d)** Correlation network diagram detailing the associations between the expression levels of the five core genes and various immune cell infiltrating fractions. **(e)** Heatmap demonstrating the Spearman correlations between the identified 6PPD-Q-associated core genes (*MAPKAPK2, CFB, PLIN2, NR1H4*, and *ANXA5*) and specific immune cell types.

To investigate the relationship between immune cell composition and UC pathogenesis, the CIBERSORT algorithm was employed to evaluate the immune cell infiltration profiles in UC mucosal lesions compared to healthy controls. The results revealed significant differences in the abundance of multiple immune cell types, including naive B cells, plasma cells, activated memory CD4+ T cells, follicular helper T cells, regulatory T cells (Tregs), resting NK cells, macrophages (M0, M1, M2), resting and activated dendritic cells, resting and activated mast cells, and neutrophils (**Figures 4a, b**).

Correlation analysis among the immune cell subsets highlighted significant interaction patterns (**Figure 4c**). Specifically, a coordinated pro-inflammatory module comprising activated NK cells, activated memory CD4+ T cells, M1 macrophages, and neutrophils exhibited strong positive correlations with each other. In contrast, these pro-inflammatory effector cells were negatively correlated with M2 macrophages and resting mast cells, suggesting a critical shift from regulatory/repair phenotypes toward a sustained inflammatory microenvironment.

Furthermore, gene-immune cell correlation analysis revealed two distinct interaction patterns among the core targets, which perfectly aligned with their differential expression trends in UC. Specifically, the upregulated pathogenic targets(*MAPKAPK2, CFB*, and *ANXA5*)exhibited strong positive correlations with pro-inflammatory and effector immune cells (e.g., M0/M1 macrophages, neutrophils, and activated mast cells) while showing negative correlations with immunosuppressive or quiescent subpopulations (e.g., M2 macrophages and resting mast cells). Conversely, the downregulated protective targets(*PLIN2* and *NR1H4*)displayed the exact opposite trend, correlating negatively with inflammatory effectors and positively with resting or regulatory subsets. These findings strongly suggest that the dysregulation of these core targets—whether through pathogenic elevation or the loss of protective suppression—is intricately linked to enhanced inflammatory immune infiltration and the disruption of local immune homeostasis in UC (**Figure 4d, e**).

### 3.7 Single-cell sequencing analysis of core genes and *in silico perturbation* validation

**Figure 5:**
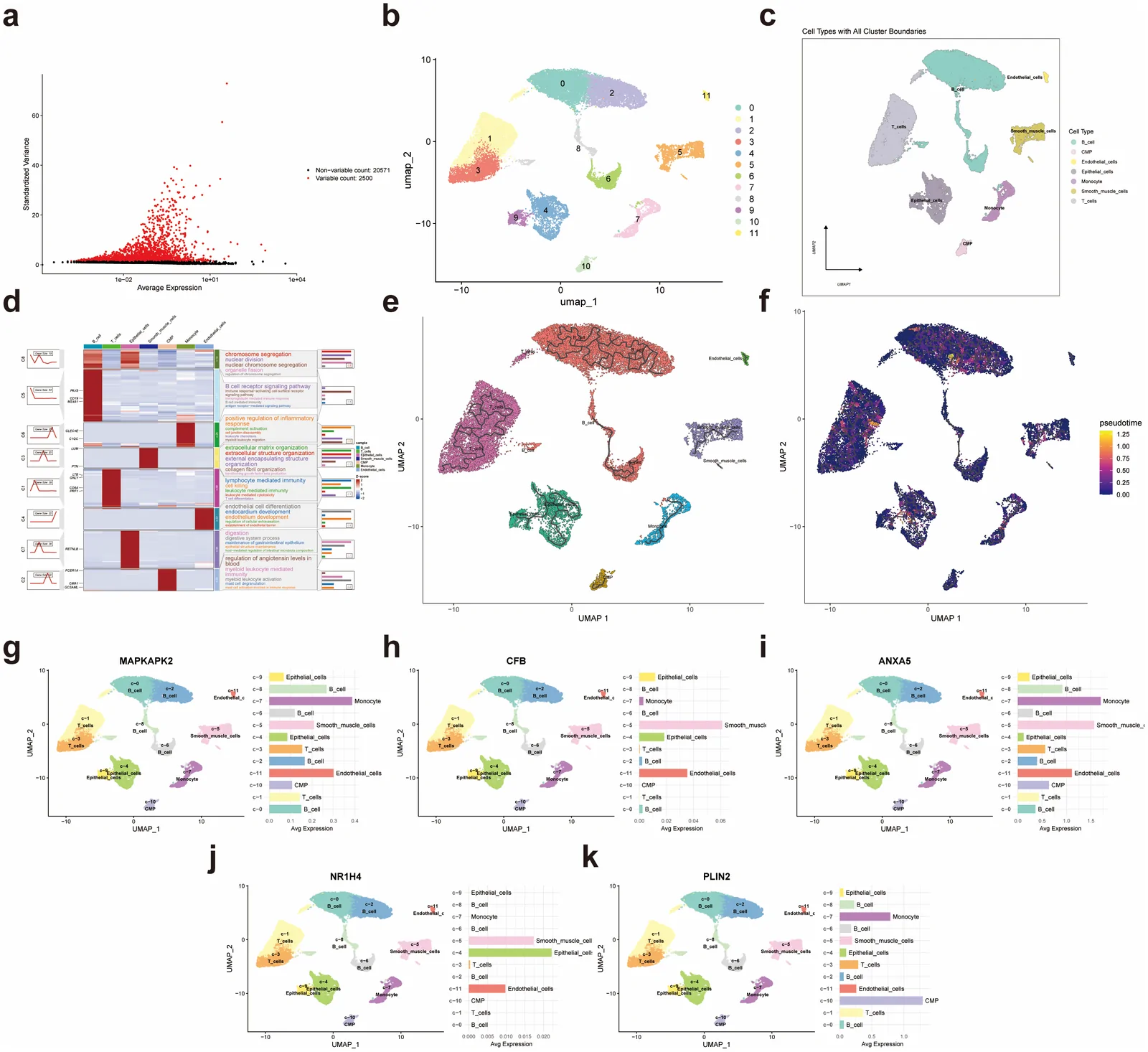
Single-cell transcriptomic analysis and pseudotime trajectory of key regulatory genes in UC **(a)** Scatter plot illustrating the relationship between average gene expression and standardized variance. Red dots represent the 2,500 highly variable genes, while black dots denote the 20,571 non-variable genes. **(b)** Uniform Manifold Approximation and Projection (UMAP) visualization of unsupervised cell clustering, with distinct colors and numerical identifiers denoting individual clusters. **(c)** UMAP plot displaying the annotation of the seven distinct cell types. **(d)** Heatmap illustrating the expression patterns of marker genes across the seven identified cell types, accompanied by functional enrichment analysis. **(e)** UMAP trajectory plot of single cells from colon samples, color-coded by the seven annotated cell types to illustrate lineage continuity. **(f)** Single-cell pseudotime trajectory analysis, with the color gradient indicating the continuous transition of cellular states from early (low values, dark blue) to late (high values, yellow) stages. **(g-k)** UMAP feature plots displaying the spatial expression patterns of the five core genes (*MAPKAPK2, CFB, ANXA5, NR1H4*, and *PLIN2*), with accompanying bar charts on the right quantifying their average expression levels across the distinct cell subpopulations.

Single-cell RNA sequencing (scRNA-seq) data from UC tissue samples underwent rigorous quality control to exclude low-quality cells and technical noise. Subsequently, 2,500 highly variable genes were selected based on gene expression variability for downstream analysis (**Figure 5a**). We first performed unsupervised clustering analysis and selected an optimal clustering resolution (**Supplementary Figure 1**). Using Uniform Manifold Approximation and Projection (UMAP) to resolve cellular heterogeneity, we successfully annotated seven distinct cell types, encompassing both immune and structural cell populations (**Figures 5b, c**). Comprehensive functional enrichment analysis further characterized these specific cell types based on their respective marker genes (**Figure 5d**).

Exploratory single-cell trajectory analysis organized multiple cell populations from the colon tissue along an inferred principal graph in low-dimensional transcriptomic space. Common myeloid progenitors (CMPs), monocytes, epithelial cells, smooth muscle cells, endothelial cells, and B/T cells occupied relatively distinct regions of the graph, reflecting differences in their transcriptional states (**Figure 5e**). The inferred pseudotime values were relatively low in the CMP and monocyte regions, whereas epithelial, smooth muscle, endothelial, B-cell, and T-cell populations occupied relatively higher positions along the computational ordering, with the highest values observed in the B- and T-cell regions (**Figure 5f**). Because these populations arise from distinct developmental lineages, the inferred ordering was interpreted solely as a descriptive representation of their relative positions in transcriptomic state space and not as evidence of lineage continuity, chronological progression, activation cascades, or direct transition from one cell population to another.

We further performed cell trajectory and pseudotime analysis on the five core genes individually (**Supplementary Figure 2a-j**). Cell type-specific expression profiling of the *MAPKAPK2, CFB, PLIN2, NR1H4*, and *ANXA5* genes revealed distinct localization patterns Specifically, *MAPKAPK2* and *ANXA5* were predominantly expressed in monocytes, *CFB* primarily in smooth muscle cells, *NR1H4* mainly in epithelial cells, while *PLIN2* showed marked upregulation in CMPs (**Figures 5g-k**). These dynamic expression signatures indicate that 6PPD-Q may mediate its immunoregulatory effects by modulating inflammatory and metabolic pathways across key cellular subpopulations—including mononuclear/myeloid cells, vascular smooth muscle cells, and intestinal epithelial cells—thereby shaping and amplifying the unique inflammatory microenvironment of UC.

**Figure 6:**
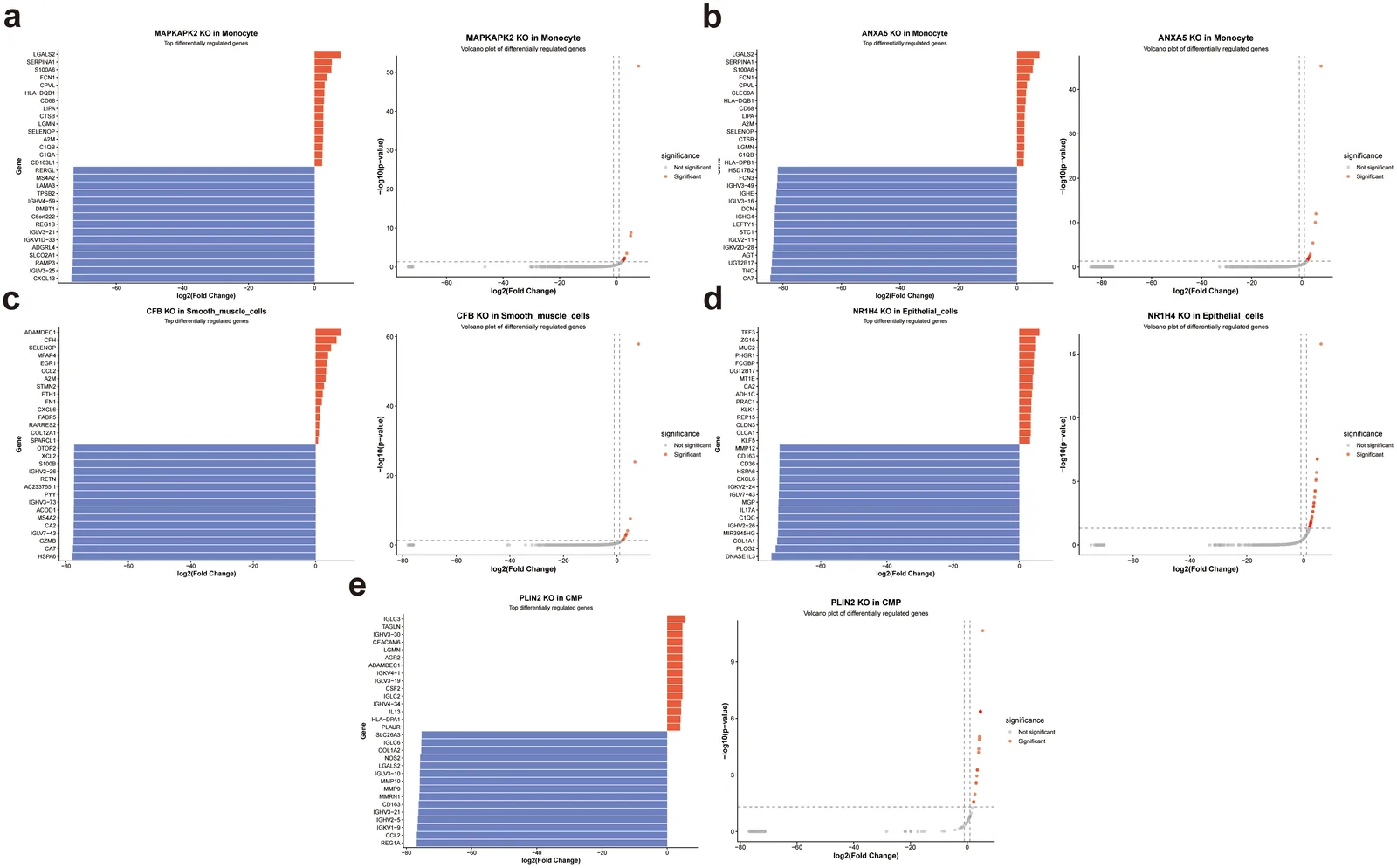
Predicted downstream network alterations following in silico perturbation of the five prioritized genes in their corresponding highly expressed cell populations. **(a)** *in silico perturbation* of MAPKAPK2 in monocytes. **(b)** *in silico perturbation* of ANXA5 in monocytes. **(c)** *in silico perturbation* of CFB in smooth muscle cells. **(d)** *in silico perturbation* of NR1H4 in epithelial cells. **(e)** *in silico perturbation* of PLIN2 in common myeloid progenitors (CMPs).

To further evaluate the biological downstream effects of these key targets, we conducted *in silico* (virtual) perturbation experiments on the five core genes within their respective highly-expressed cell clusters: *MAPKAPK2* and *ANXA5* in UC monocytes, *CFB* in smooth muscle cells, *NR1H4* in epithelial cells, and *PLIN2* in CMPs. The results indicated that the disrupted downstream genes were predominantly enriched in monocyte/myeloid inflammation and complement-phagocytosis-related components (e.g., *IGLC3, LGALS2, FCN1*), as well as chemokines and extracellular matrix (ECM) remodeling genes in vascular smooth muscle cells (e.g., *CCL2, CXCL6, RARRES2*). This points to a profound reprogramming of inflammation amplification and tissue remodeling pathways. Concurrently, epithelial barrier and mucus-related genes (e.g., *ADAMDEC1, MUC2, TFF3*) and metabolic detoxification factors (e.g., *ADH1C, UGT2B17, MT1E*) in epithelial cells were also significantly disrupted, suggesting potential associations with epithelial mucus-related functions and local metabolic processes. (**Figures 6a-e**). Collectively, these in silico perturbation findings suggest potential regulatory associations of the five prioritized genes within the UC cellular microenvironment.

### 3.8 molecular docking analysis of 6PPD-Q with core target proteins

**Figure 7:**
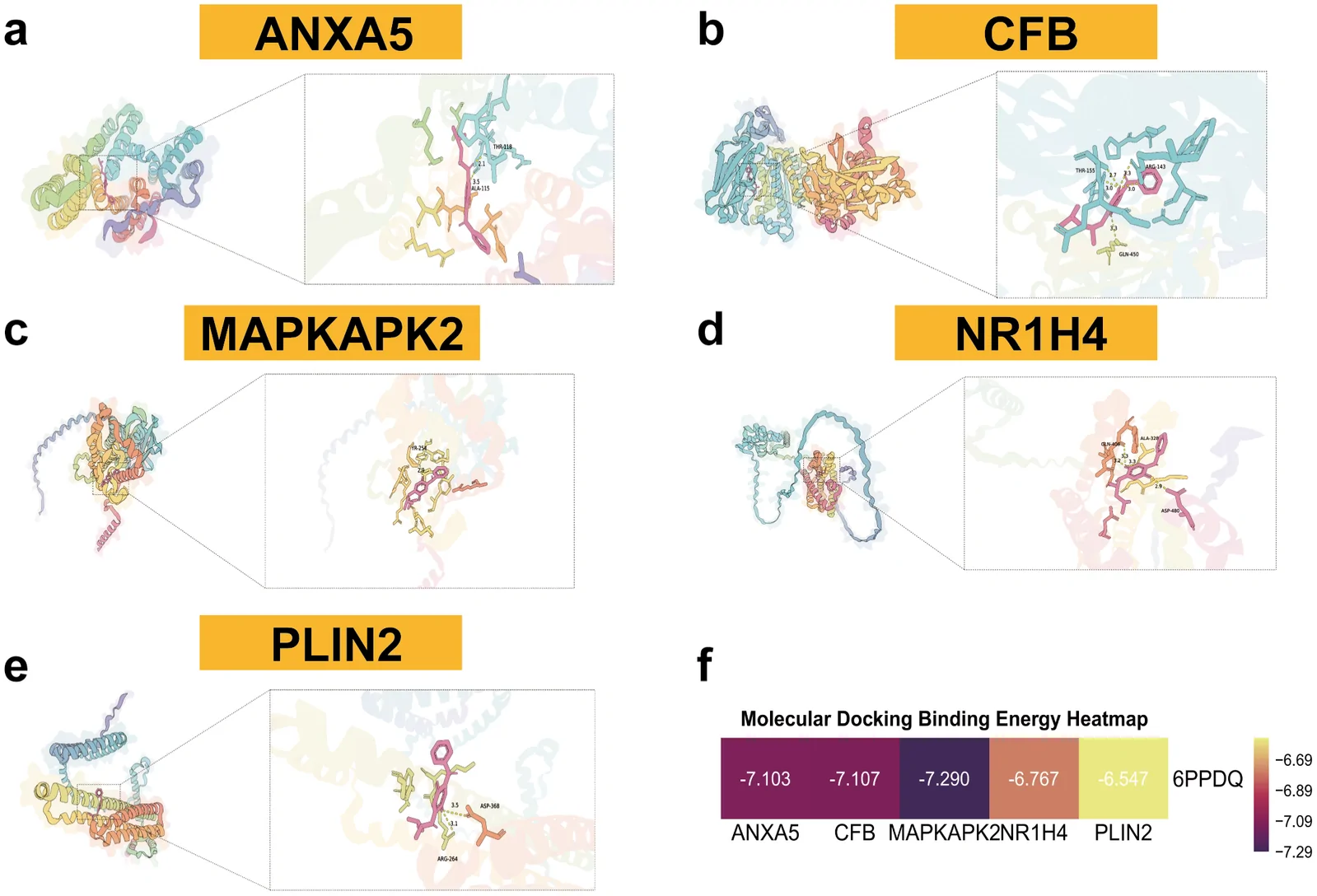
Predicted molecular docking conformations and docking scores of 6PPD-Q with the five prioritized proteins. **(a)** Predicted binding pose of 6PPD-Q with annexin A5 (ANXA5). **(b)** Predicted binding site and interacting residues of 6PPD-Q with complement factor B (CFB). **(c)** Predicted docking conformation of 6PPD-Q with MAP kinase-activated protein kinase 2 (MAPKAPK2). **(d)** Predicted interaction model of 6PPD-Q with nuclear receptor subfamily 1 group H member 4 (NR1H4). **(e)** Predicted binding pose of 6PPD-Q with perilipin-2 (PLIN2). **(f)** Heatmap summarizing the predicted molecular docking scores of 6PPD-Q with the five proteins, expressed in kcal/mol. More negative values represent more favorable predicted docking scores under the selected computational conditions.

Within the framework of computational toxicology, molecular docking studies were conducted to investigate the potential binding interactions between the environmental pollutant 6PPD-Q and the predicted UC-associated target proteins. AutoDock Vina yielded predicted docking scores of −7.103, −7.107, −7.290, −6.767, and −6.547 kcal/mol for ANXA5, CFB, MAPKAPK2, NR1H4, and PLIN2, respectively (**Figure 7**). Among the five candidate proteins, MAPKAPK2 showed the most favorable predicted docking score; however, the numerical differences from CFB and ANXA5 were modest and were insufficient to identify MAPKAPK2 as the unique primary or direct target of 6PPD-Q. MAPKAPK2 was selected as a representative prioritized candidate for exploratory molecular dynamics analysis based on the convergence of multiple computational, transcriptomic, and *in vivo* transcriptional findings.

### 3.9 Molecular dynamics simulation and combined free energy evaluation

Although molecular docking aids in elucidating preliminary binding patterns between ligands and receptors, its static nature struggles to reflect the dynamic behavior of proteins in near-physiological environments. Therefore, to systematically evaluate the stability and conformational changes of the 6PPD-Q–MAPKAPK2 complex, we conducted a 100-nanosecond (ns) molecular dynamics (MD) simulation.

**Figure 8:**
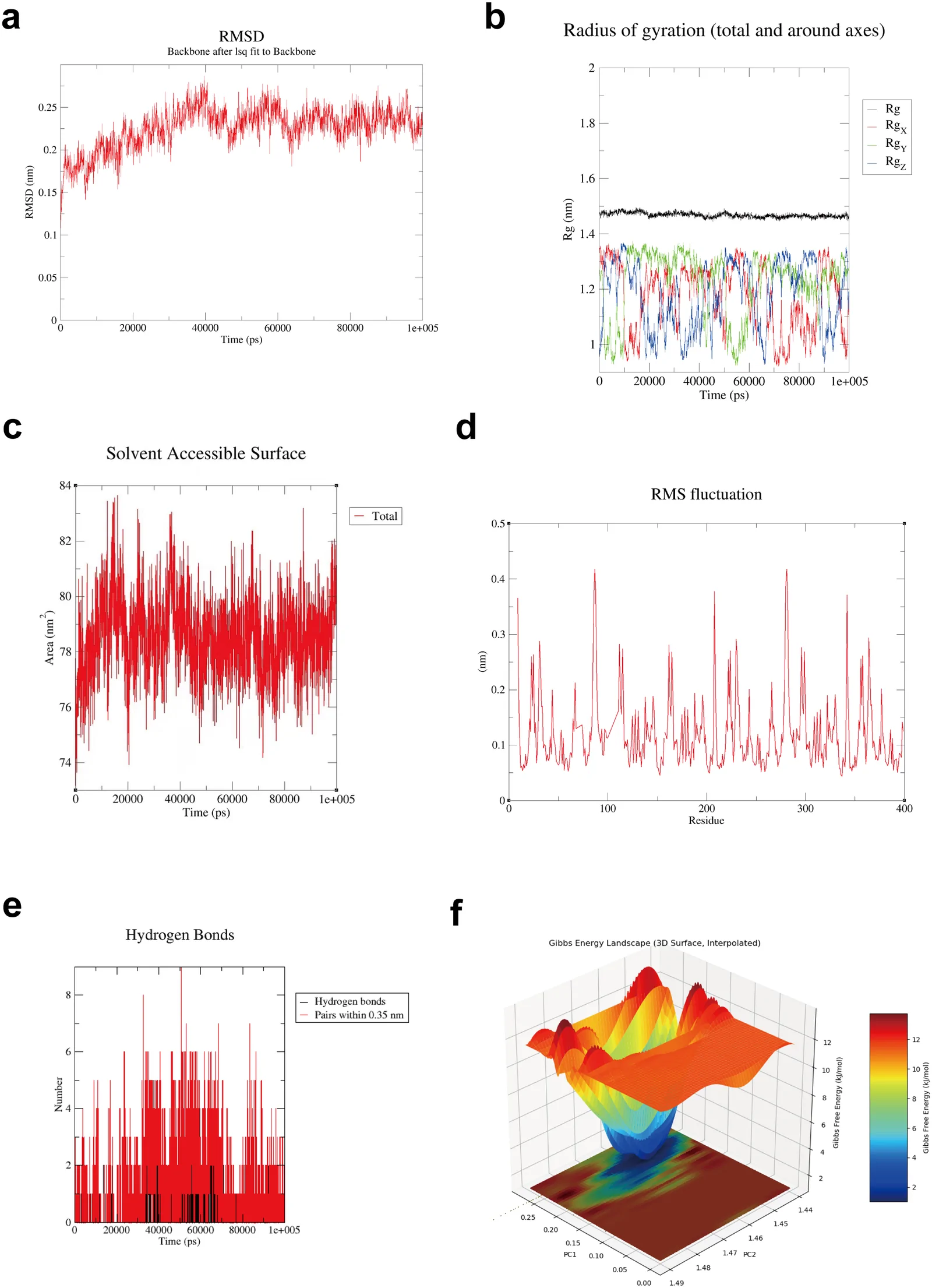
Molecular dynamics simulations of the 6PPD-Q–MAP kinase-activated protein kinase 2 (MAPKAPK2) complex to evaluate its structural stability and interaction characteristics. **(a)** Time-dependent root-mean-square deviation (RMSD) curve characterizing the conformational stability of the 6PPD-Q–MAPKAPK2 complex throughout the 100 ns simulation. **(b)** Radius of gyration (Rg) over time, reflecting the overall compactness and structural integrity of the complex during the simulation. **(c)** Solvent-accessible surface area (SASA) trajectory, illustrating the dynamic changes in surface exposure of the complex over time. **(d)** Root-mean-square fluctuation (RMSF) analysis, revealing the distribution of local flexibility among MAPKAPK2 amino acid residues in the 6PPD-Q-bound state. **(e)** Temporal evolution of hydrogen bond formation between 6PPD-Q and MAPKAPK2, indicating the persistence and stability of ligand-protein interactions. **(f)** Gibbs free energy landscape (FEL) constructed via principal component analysis, illustrating a predominant low-energy conformational region sampled by the 6PPD-Q–MAPKAPK2 complex under the selected simulation conditions.

As depicted in **Figure 8a**, the Root Mean Square Deviation (RMSD) results indicate that the protein scaffold and ligand undergo rapid conformational adjustments during the initial phase before converging. Subsequently, they maintain fluctuations within a consistently low and narrow range, suggesting that the complex reaches equilibrium and sustains stable interactions during the latter half of the simulation. Similarly, the radius of gyration (Rg) (**Figure 8b**) fluctuates slightly around a fundamentally constant average value throughout the trajectory, with no discernible trend of sustained increase or decrease. This indicates that the protein maintains a compact overall folding state without undergoing significant conformational unfolding or collapse. Furthermore, although the solvent-accessible surface area (SASA) (**Figure 8c**) exhibited high-frequency fluctuations between 74–84 nm², it did not show a consistent upward or downward trend overall, indicating that the complex’s exposure to the solvent remained largely constant. Root Mean Square Fluctuation (RMSF) analysis (**Figure 8d**) further reveals that most residues exhibit only minor thermal fluctuations, with higher flexibility confined primarily to a few loop regions, indirectly supporting the rigidity of the complex’s backbone structure. Meanwhile, the number of hydrogen bonds between 6PPD-Q and MAPKAPK2 (**Figure 8e**) persisted throughout the simulation, indicating the presence of enduring noncovalent interactions.

Crucially, to explore the dominant conformational states during the simulation, a Gibbs Free Energy Landscape (FEL) was constructed (**Figure 8f**). The three-dimensional free-energy landscape showed a concentrated low-energy basin, indicating that the trajectory sampled a predominant low-energy conformational region under the selected simulation conditions.

Collectively, the simulation analyses suggested that the predicted 6PPD-Q–MAPKAPK2 complex maintained a relatively stable trajectory during the single 100-ns simulation under the selected computational conditions. These findings do not establish direct biological binding, functional target engagement, or reproducibility across independent trajectories.

### 3.10 *In vivo* validation in the 6PPD-Q-exposed animal model

**Figure 9:**
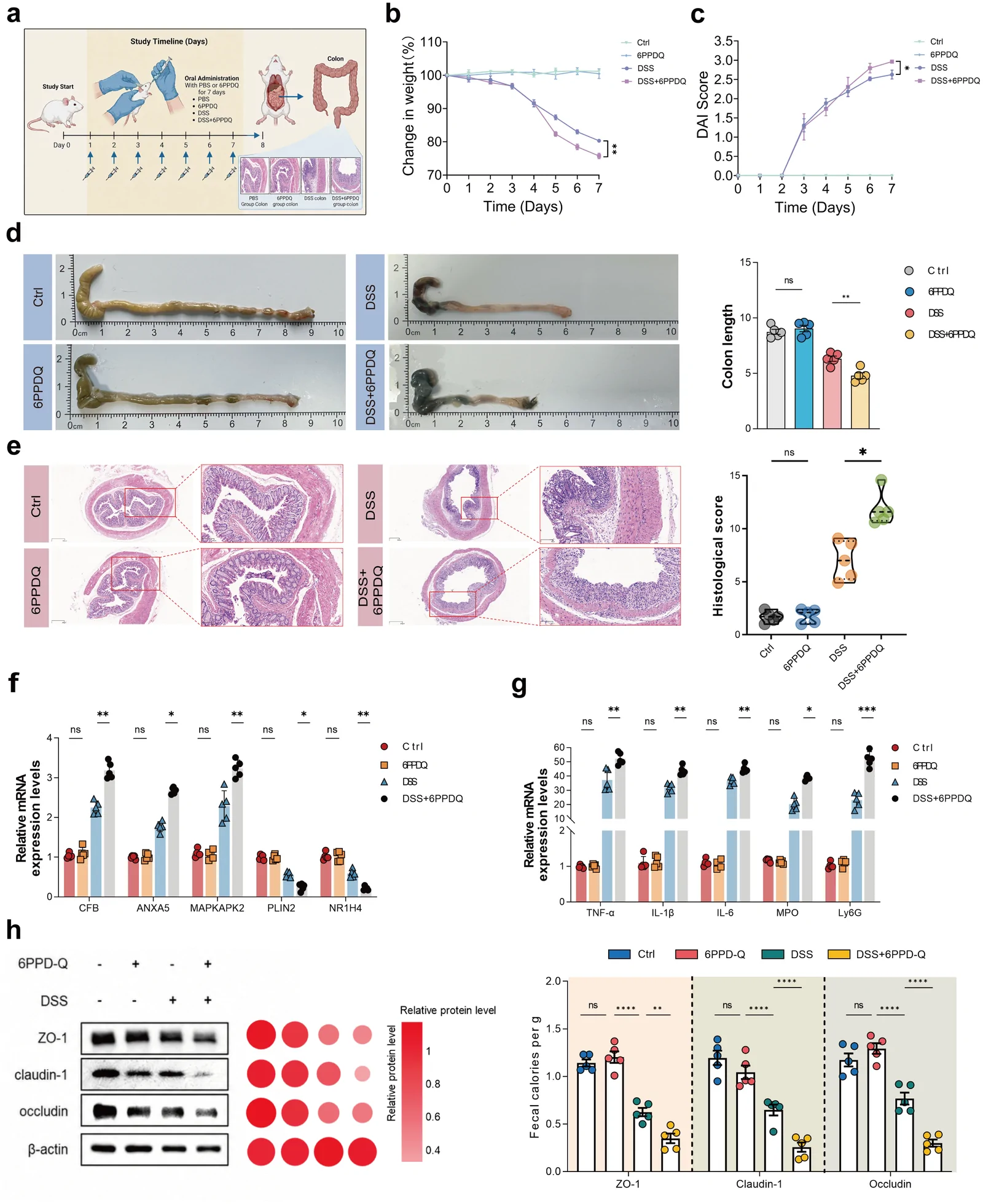
*In vivo* evaluation of the effects of 6PPD-Q on DSS-induced colitis and intestinal epithelial-barrier-associated proteins. **(a)** Experimental design and treatment timeline for the control, 6PPD-Q-alone, DSS-alone, and DSS plus 6PPD-Q groups. **(b)** Changes in body weight during the 7-day experimental period. **(c)** Disease Activity Index (DAI) scores. **(d)** Representative macroscopic colon images and quantitative analysis of colon length. **(e)** H&E stained colonic sections and histological damage scores. **(f)** Relative colonic mRNA expression levels of Cfb, Anxa5, Mapkapk2, Plin2, and Nr1h4. **(g)** Relative colonic mRNA expression levels of the inflammatory cytokine-related genes Tnf, Il1b, and Il6 and the neutrophil-associated genes Mpo and Ly6g. **(h)** Representative Western blot images and densitometric quantification of ZO-1, claudin-1, and occludin protein expression in colonic tissues. Protein expression was normalized to β-actin. Each point represents one biological replicate. Data are presented as the mean ± SD. ns, not significant; ^*^P < 0.05, ^**^P < 0.01, ^***^P < 0.001, and ****P < 0.0001.

To validate the in silico findings and evaluate the effects of 6PPD-Q on intestinal inflammation, we established a DSS-induced colitis mouse model comprising four groups: control, 6PPD-Q alone, DSS alone, and DSS plus 6PPD-Q (**Figure 9a**). Compared with the control group, short-term exposure to 6PPD-Q alone did not significantly affect body weight, Disease Activity Index (DAI), colon length, or histological damage, indicating that 6PPD-Q alone did not induce overt colitis under the present experimental conditions. In contrast, DSS administration caused progressive body-weight loss and increased DAI scores, and these changes were significantly more pronounced in the DSS + 6PPD-Q group than in the DSS-only group (**Figures 9b, c**).

Macroscopic examination further showed that DSS induced marked colon shortening, which was significantly aggravated by concurrent exposure to 0.1 mg/kg 6PPD-Q (**Figure 9d**). Consistent with these findings, H&E staining revealed inflammatory cell infiltration, crypt disruption, and mucosal injury in DSS-treated mice, whereas the DSS + 6PPD-Q group exhibited more extensive mucosal damage and significantly higher histological scores. No significant histological alterations were observed in the 6PPD-Q-alone group compared with the control group (**Figure 9e**).

At the molecular level, qRT-PCR analysis of colonic tissues showed that Cfb, Anxa5, and Mapkapk2 were upregulated following DSS administration and were further increased in the DSS + 6PPD-Q group. Conversely, Plin2 and Nr1h4 were downregulated in DSS-treated mice and showed a further reduction following combined DSS and 6PPD-Q exposure. No significant differences in the expression of these five genes were detected between the control and 6PPD-Q-alone groups (**Figure 9f**). The directions of these transcriptional changes were consistent with the bioinformatics predictions and provided additional support for prioritizing these genes as candidate targets associated with 6PPD-Q-exacerbated intestinal injury. However, these mRNA-level findings do not establish corresponding changes in protein abundance or functional activity. In addition, the colonic expression levels of the inflammatory cytokine-related genes *TNF-α, IL-1β*, and *IL-6*, together with the neutrophil-associated markers *MPO* and *Ly6G*, were markedly increased in the DSS group and further elevated in the DSS + 6PPD-Q group, whereas 6PPD-Q exposure alone produced no significant changes (**Figure 9g**).Western blot analysis further demonstrated that the colonic protein expression levels of the tight-junction proteins ZO-1, claudin-1, and occludin were significantly reduced following DSS administration. Compared with the DSS-only group, the DSS plus 6PPD-Q group showed further reductions in all three proteins, whereas no significant differences were observed between the control and 6PPD-Q-alone groups (**Figure 9h**). These findings provide protein-level evidence that 6PPD-Q aggravates DSS-associated loss of intestinal tight-junction proteins under the tested conditions. Collectively, these results indicate that short-term exposure to 0.1 mg/kg/day 6PPD-Q alone did not induce overt colitis under the present experimental conditions but significantly exacerbated DSS-induced clinical manifestations, histopathological injury, inflammatory and neutrophil-associated transcriptional responses, and loss of intestinal tight-junction proteins.

### 3.11 Construction of a proposed hypothesis-generating AOP framework

**Figure 10:**
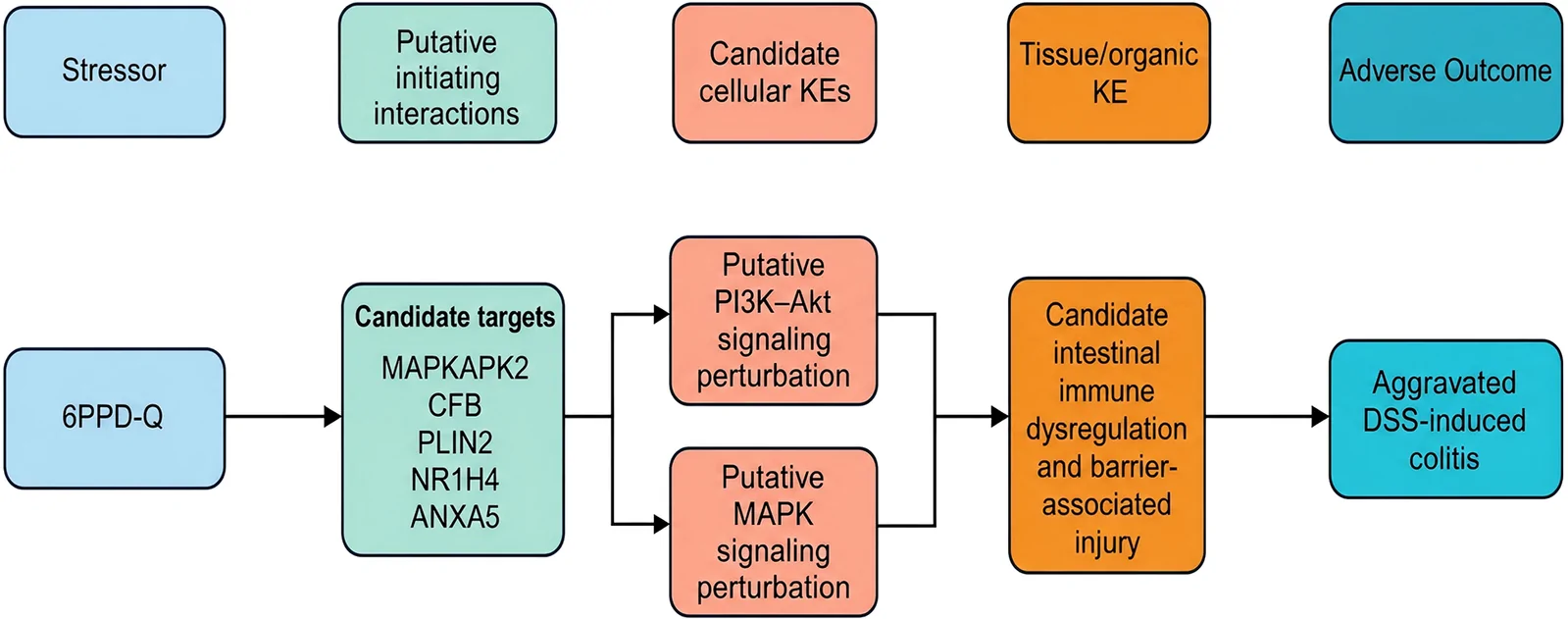
Proposed adverse outcome pathway (AOP) framework summarizing a hypothesized sequence of molecular and cellular events linking 6PPD-Q exposure to aggravated intestinal inflammation in ulcerative colitis.

By integrating the multidimensional in silico and *in vivo* evidence, we proposed an Adverse Outcome Pathway (AOP) framework to summarize a potential sequence of events linking 6PPD-Q exposure to aggravated intestinal inflammation (**Figure 10**). In this framework, 6PPD-Q is considered the environmental stressor, while its predicted interactions with the five prioritized candidate targets—*MAPKAPK2, ANXA5, CFB, NR1H4*, and *PLIN2*—are regarded as putative initiating interactions involving the five candidate targets. Perturbations of PI3K-Akt and MAPK signaling, intestinal immune dysregulation, and epithelial-barrier impairment are proposed as candidate key events that may contribute to the adverse outcome of aggravated colitis. This framework organizes the available computational, transcriptional, and phenotypic evidence into a testable mechanistic hypothesis rather than establishing a definitive causal pathway.

## 4 Discussion

Ulcerative colitis (UC) is a chronic, immune-mediated inflammatory bowel disease with a multifactorial etiology involving the interaction of genetic susceptibility and environmental exposure. Environmental pollutants, particularly emerging organic pollutants, are increasingly recognized as contributing to the onset and persistent course of ulcerative colitis, though their toxicological mechanisms remain poorly understood. This study focuses on 6PPD-Q—an environmental pollutant derived from tire wear—and employs a comprehensive approach integrating network toxicology, machine learning, transcriptomic validation, molecular docking, kinetic modeling, and animal model validation to systematically elucidate its potential pathogenic role in the onset and progression of ulcerative colitis (UC).

Both epidemiological and experimental studies indicate that multiple environmental pollutants are closely associated with the onset and exacerbation of UC (14, 15). Air pollutants such as fine particulate matter (PM2.5), ozone, nitrogen dioxide, and polycyclic aromatic hydrocarbons can amplify colonic inflammatory responses by inducing oxidative stress and mucosal barrier damage, thereby activating both the innate and adaptive immune systems of the intestinal mucosa (21, 22). Certain metals and organic pollutants can also alter the composition of the gut microbiota and its metabolites, disrupting the homeostasis of the “microbiota-epithelium-immune” axis. This promotes macrophage and neutrophil infiltration, T-cell lineage imbalance, and diminished mucosal repair capacity (23, 24). Overall, environmental pollutants serve as both significant external triggers for disease recurrence in UC patients and as key risk factors driving the progression of chronic inflammation toward fibrosis and even malignant transformation.

Available evidence indicates that 6PPD-Q is widely detected in outdoor air, indoor dust, soil, and water bodies, and is closely associated with immune dysregulation and pro-inflammatory responses (25). Animal and cellular experimental evidence indicates that 6PPD-Q exposure can induce apoptosis and impaired barrier function by activating key signaling pathways such as PI3K-Akt. This is accompanied by the upregulation of immune-related genes and increased myeloid cell infiltration, leading to adverse outcomes including ovarian damage and hepatotoxicity (10, 26). Therefore, these findings underscore the immunotoxic and proinflammatory potential of 6PPD-Q, suggesting its possible role in the pathogenesis and progression of immune-mediated diseases.

Using a network toxicology approach, we identified 158 candidate genes potentially associated with 6PPD-Q-exacerbated ulcerative colitis. Functional enrichment analysis of the overlapping candidate genes highlighted immune- and inflammation-related pathways, including TNF, IL-17, Toll-like receptor, MAPK, and PI3K-Akt signaling. These findings represent predicted pathway associations rather than experimentally demonstrated pathway activation by 6PPD-Q. It also enriched pathways associated with Th17 cell differentiation, cytokine–receptor interactions, and various infection- and tumor-related processes, suggesting its broad regulatory effects on the mucosal immune network. At the level of immune cells, we found that 6PPD-Q exposure was closely associated with increased infiltration of pro-inflammatory subsets, including activated dendritic cells (DCs), M1 macrophages, neutrophils, activated mast cells, and Th17-related T cells. Conversely, immunosuppressive or homeostatic subsets, such as Tregs, M2 macrophages, and resting NK cells, were relatively reduced. This indicates that 6PPD-Q tends to remodel the intestinal microenvironment toward a pro-inflammatory dominant immune pattern.

Through LASSO and random forest machine learning algorithms, we identified five core regulatory genes—*MAPKAPK2, ANXA5, CFB, NR1H4*, and *PLIN2*—from the overlapping 6PPD-Q and UC targets. Importantly, these genes exhibited distinct dysregulation patterns in UC tissues (specifically, the significant upregulation of *MAPKAPK2, ANXA5*, and *CFB*, alongside the notable downregulation of *NR1H4* and *PLIN2*), and demonstrated robust diagnostic performance in both the training and validation cohorts (AUC values approaching or exceeding 0.90). Immune infiltration correlation analysis revealed that these core targets were generally correlated with the recruitment of pro-inflammatory immune subpopulations while negatively correlated with immunoregulatory populations. These findings suggest that 6PPD-Q disrupts intestinal immune homeostasis at multiple cellular levels by driving the activation of inflammatory cells while simultaneously impairing immunosuppression and barrier protection mechanisms. Further integrating single-cell transcriptomics and simulated perturbation analysis, we revealed the cell-type-specific roles of these targets. 6PPD-Q modulates inflammatory and metabolic hub genes, notably amplifying inflammatory cytokine production, complement activation, and ECM remodeling pathways in monocytes/macrophages (via *MAPKAPK2* and *ANXA5*) and vascular smooth muscle cells (via *CFB*). Concurrently, it impairs the intestinal epithelial barrier, bile acid, and lipid metabolism primarily via the *NR1H4*-regulated FXR axis. This suggests that 6PPD-Q delivers a complex, dual pathogenic effects: triggering immune cell hyperactivation while dismantling intrinsic epithelial defenses.

Molecular docking yielded favorable predicted scores for 6PPD-Q with the five prioritized proteins, with MAPKAPK2 showing the most favorable score of −7.290 kcal/mol. Subsequent molecular dynamics simulation supported a relatively stable predicted trajectory of the 6PPD-Q–MAPKAPK2 complex under the selected computational conditions. These structural analyses support the plausibility of a potential interaction but do not independently establish direct physical binding, functional target engagement, or the role of MAPKAPK2 as a confirmed molecular initiating event.

*In vivo* experimental data further supported the aggravating effect of 6PPD-Q under intestinal inflammatory conditions. The inclusion of a standalone 6PPD-Q exposure group showed that short-term oral administration of 0.1 mg/kg 6PPD-Q alone did not significantly alter body weight, DAI score, colon length, histological damage, or the colonic expression of the five core genes and inflammatory markers. This finding suggests that 6PPD-Q alone was insufficient to induce overt colitis under the present experimental conditions.

In contrast, concurrent 6PPD-Q exposure significantly aggravated DSS-induced body-weight loss, disease activity, colon shortening, and histopathological injury. The DSS + 6PPD-Q group also exhibited greater upregulation of *Cfb, Anxa5*, and *Mapkapk2*, further downregulation of *Plin2* and *Nr1h4*, and increased expression of the inflammatory cytokine-related genes Tnf, Il1b, and Il6 and the neutrophil-associated markers Mpo and Ly6g compared with the DSS-only group. These findings indicate that the intestinal effects of 6PPD-Q became more evident in an inflammation-susceptible environment and support its role as an aggravating environmental factor rather than an independent inducer of overt colitis under the tested conditions.

These findings should be interpreted in the context of previous studies on the intestinal toxicity of 6PPD-Q. Previous experiments conducted in otherwise healthy mice reported that oral 6PPD-Q exposure predominantly affected the jejunum and ileum, with intestinal-barrier disruption and cannabinoid receptor-associated inflammatory responses, whereas marked colonic injury was relatively limited (27). These observations are not necessarily inconsistent with the present findings. In our study, DSS had already compromised colonic epithelial integrity and mucosal immune homeostasis, potentially rendering the colon more susceptible to an additional environmental stressor. Accordingly, 6PPD-Q may exert limited short-term effects on an intact colon while amplifying inflammatory and histopathological injury in a pre-existing inflammatory environment. The candidate MAPKAPK2/PI3K-Akt/MAPK-related processes proposed in the present study should therefore be considered complementary to, rather than replacements for, previously reported cannabinoid receptor- and epithelial-barrier-associated mechanisms.

Based on these multidimensional findings, we proposed an AOP framework to organize a hypothesized sequence linking 6PPD-Q exposure with aggravated intestinal inflammation. In this framework, the five prioritized targets represent putative initiating interactions involving the five candidate targets, whereas predicted perturbations of PI3K-Akt/MAPK signaling, intestinal immune dysregulation, and epithelial-barrier impairment are considered candidate key events that may contribute to aggravated colitis. This framework provides a structured and testable hypothesis rather than definitive evidence of a complete causal sequence.

Although the proposed AOP provides a coherent framework linking 6PPD-Q exposure to aggravated intestinal inflammation, several evidence gaps should be addressed before its application in regulatory decision-making. First, quantitative key-event relationships remain insufficiently characterized because the present *in vivo* experiment evaluated a single proof-of-concept dose over a short exposure period. Multi-dose and time-course studies, combined with measurements of internal 6PPD-Q concentrations and metabolites, are required to establish dose–response and temporal concordance across molecular target perturbation, PI3K-Akt/MAPK signaling, inflammatory responses, epithelial injury, and colitis severity. Second, the precise molecular initiating event and the essentiality of the proposed key events require further experimental validation. Direct target-engagement assays and genetic or pharmacological intervention studies are needed to determine whether MAPKAPK2 and the associated signaling pathways are necessary for the aggravating effects of 6PPD-Q. Third, the human relevance and domain of applicability of this AOP should be evaluated using human intestinal epithelial and immune-cell systems, intestinal organoids, clinically derived UC tissues, and toxicokinetic or physiologically based pharmacokinetic approaches. Independent validation across sex, exposure duration, and experimental platforms will further strengthen the weight of evidence and support the future regulatory applicability of this AOP.

The innovation of this study lies in the integration of network toxicology, machine learning, single-cell analysis, structural biology, and *in vivo* validation to comprehensively map the toxicological mechanisms of 6PPD-Q in UC. This integrative strategy provides a basis for generating testable hypotheses regarding the potential intestinal effects of 6PPD-Q under inflammatory conditions. Although this study integrates computational prediction with *in vivo* validation to investigate the potential mechanisms by which 6PPD-Q exacerbates intestinal inflammation, several limitations should be acknowledged. First, the *in vivo* experiment evaluated only a single proof-of-concept dose of 6PPD-Q over a short 7-day exposure period. Consequently, dose–response relationships, chronic exposure effects, sex-dependent responses, and potential delayed toxicity were not determined. Moreover, internal 6PPD-Q concentrations, metabolites, and toxicokinetic parameters were not measured; therefore, the administered dose should not be interpreted as a direct human-equivalent exposure level. In addition, the animal experiment focused primarily on colonic injury. Other organs, hematological parameters, serum biochemical indices, circulating cytokines, and systemic inflammatory markers were not systematically evaluated. Therefore, potential extraintestinal toxicity or systemic inflammatory effects of 6PPD-Q cannot be excluded. Finally, experimental validation of the five prioritized candidate targets was mainly conducted at the transcriptional level. Although the tight-junction proteins ZO-1, claudin-1, and occludin were evaluated by Western blotting, the protein abundance, cellular localization, and phosphorylation status of MAPKAPK2 and the other prioritized targets were not directly assessed. Activation of the p38/MAPKAPK2 and PI3K-Akt signaling pathways was also not evaluated using pathway-protein, phosphorylation, kinase-activity, or functional-intervention assays. Furthermore, intestinal permeability and immune-cell-subset-specific infiltration were not directly measured. Therefore, MAPKAPK2 and the PI3K-Akt/MAPK-related processes should be regarded as prioritized candidate mechanisms rather than experimentally established causal mediators. Nevertheless, these findings mathematically prioritize a highly relevant core gene panel (*MAPKAPK2, CFB, PLIN2, NR1H4*, and *ANXA5*), whose clinical applicability requires further prospective validation in multicenter cohorts.

## 5 Conclusion

This study systematically investigated the toxicological mechanisms of 6PPD-Q-exacerbated ulcerative colitis (UC) by integrating computational predictions with experimental validation. We screened 158 candidate targets and prioritized five core regulatory genes (*MAPKAPK2, ANXA5, CFB, NR1H4*, and *PLIN2*) through machine learning algorithms. Molecular docking and molecular dynamics simulations supported that 6PPD-Q showed plausible predicted interactions with the prioritized targets. Crucially, the DSS-induced UC mouse model *in vivo* validated the severe histopathological damage to colonic tissues and the distinctly dysregulated expression of these core genes, highly consistent with our bioinformatics predictions. Finally, a proposed AOP framework was developed to organize the available evidence linking 6PPD-Q exposure with candidate molecular events, intestinal immune dysregulation, epithelial-barrier injury, and aggravated colitis. This framework provides testable mechanistic hypotheses that require further validation through dose–response, temporal, protein-level, and functional intervention studies. These findings strongly suggest that the emerging environmental pollutant 6PPD-Q drives UC pathological processes through complex, multi-target mechanisms, providing a robust theoretical framework and potential therapeutic intervention targets for subsequent toxicological research.

## Data Availability

The processed data and cleaned, annotated article-specific analysis code are available from the corresponding author upon reasonable request.
